# Bone single photon emission computed tomography (SPECT) in a patient with Pancoast tumor: a case report

**DOI:** 10.1590/S1516-31802010000400013

**Published:** 2010-07-01

**Authors:** Hamid Javadi, Mehdi Mogharrabi, Isa Neshandar Asli, Babak Shafiei, Mehrzad Bahtoee, Mohammad Seyedabadi, Iraj Nabipour, Majid Assadi

**Affiliations:** I MD. Head of Department of Nuclear Medicine, 5^th^ Azar Hospital, Golestan University of Medical Sciences, Gorgan, Iran.; II MD. Assistant professor of Nuclear Medicine, Department of Nuclear Medicine, 5^th^ Azar Hospital, Golestan University of Medical Sciences, Gorgan, Iran.; III MD. Head of Department of Nuclear Medicine, Taleghani Hospital, Shaheed Beheshti University of Medical Sciences, Tehran, Iran.; IV Assistant professor of Nuclear Medicine, Taleghani Hospital, Shaheed Beheshti University of Medical Sciences, Tehran, Iran.; V MD. Assistant professor of Internal Medicine, Tropical and Geographical Research Center, Persian Gulf Biomedical Sciences Institute, Bushehr University of Medical Sciences, Bushehr, Iran.; VI PhD. Pharmacologist and researcher in the Bushehr Research Center for Nuclear Medicine, Persian Gulf Biomedical Sciences Institute, Bushehr University of Medical Sciences, Bushehr, Iran.; VII MD. Professor of Internal Medicine, Tropical and Geographical Research Center, Persian Gulf Biomedical Sciences Institute, Bushehr University of Medical Sciences, Bushehr, Iran.; VIII MD. Head of Bushehr Research Center for Nuclear Medicine, Persian Gulf Biomedical Sciences Institute, Bushehr University of Medical Sciences, Bushehr, Iran.

**Keywords:** Carcinoma, non-small-cell lung, Pancoast syndrome, Technetium Tc 99m medronate, Tomography, emission-computed, single-photon, Magnetic resonance imaging, Carcinoma pulmonar de células não pequenas, Síndrome de Pancoast, Medronato de tecnécio Tc 99m, Tomografia computadorizada de emissão de fóton único, Imagem por ressonância magnética

## Abstract

**CONTEXT:**

Non-small cell lung carcinomas (NSCLCs) of the superior sulcus are considered to be the most challenging type of malignant thoracic disease. In this disease, neoplasms originating mostly from the extreme apex of the lung expand to the chest wall and thoracic inlet structures. Multiple imaging procedures have been applied to identify tumors and to stage and predict tumor resectability in surgical operations. Clinical examinations to localize pain complaints in shoulders and down the arms, and to screen for Horner’s syndrome and abnormalities seen in paraclinical assessments, have been applied extensively for differential diagnosis of superior sulcus tumors. Although several types of imaging have been utilized for diagnosing and staging Pancoast tumors, there have been almost no reports on the efficiency of whole-body bone scans (WBBS) for detecting the level of abnormality in cases of superior sulcus tumors.

**CASE REPORT:**

We describe a case of Pancoast tumor in which technetium-99m methylene diphosphonate (Tc-99m MDP) bone single-photon emission-computed tomography (SPECT) was able to accurately detect multiple areas of abnormality in the vertebrae and ribs. In describing this case, we stress the clinical and diagnostic points, in the hope of stimulating a higher degree of suspicion and thereby facilitating appropriate diagnosis and treatment. From the results of this study, further clinical trials to evaluate the potential of SPECT as an efficient imaging tool for the work-up on cases of Pancoast tumor are recommended.

## RESUMO

**CONTEXTO:**

Carcinomas pulmonares de células não pequenas (NSCLCs) do sulco superior são considerados como o maior desafio nos tumores malignos torácicos. Nesta doença, as neoplasias se originam principalmente do ápice do pulmão e se expandem pela parede e pelas estruturas torácicas. Diversas técnicas de imagem têm sido utilizadas para identificar e estagiar os tumores, permitindo um prognóstico para a sua ressecção em procedimentos cirúrgicos. O exame clínico tem sido bastante usado para diagnóstico diferencial de tumores do sulco superior nas queixas de dores no ombro, síndrome de Horner e anormalidades observadas em exames paraclínicos. Embora diversos tipos de exames de imagem sejam utilizados para diagnóstico e estadiamento de tumores de Pancoast, praticamente não existem relatos sobre a eficácia do exame ósseo do corpo todo (WBBS) na detecção do nível de anormalidade em casos de tumores do sulco superior.

**RELATO DE CASO:**

Descrevemos um caso de tumor de Pancoast, em que foi realizada a tomografia computadorizada óssea por emissão de fóton único (SPECT) com metileno difosfonato de tecnécio-99 (Tc-99m MDP). Este exame foi capaz de detectar com acurácia as múltiplas áreas de anormalidade em vértebras e costelas. Ao descrever este caso, os autores ressaltam os pontos clínicos e diagnósticos, esperando estimular maior grau de suspeita, facilitando assim o diagnóstico e tratamento apropriados. A partir dos resultados deste trabalho, outros estudos clínicos podem avaliar o potencial do SPECT como uma ferramenta eficiente de imagem a ser recomendada na investigação de casos de tumor de Pancoast.

## INTRODUCTION

Pancoast tumors, first described by Pancoast and Tobias,^1-3^ account for less than 5% of non-small cell lung carcinomas (NSCLCs).^4^ They are a rare subgroup of neoplasms that emanate mostly from the apex of the lung and expand to the chest wall and brachial plexus.^5^ The involvement of subclavian vessels, stellate ganglions, mediastinal lymph nodes and adjacent vertebral bodies demands more sophisticated instruments for surgical treatment and may worsen the survival rate.^4^ The etiology of the disease includes either primary thoracic carcinomas, or it may be secondary to metastatic or hematological neoplasms and a variety of infections.^1^

Clinical examinations to localize the cause of pain complaints in shoulders and down the arms, and to screen for Horner’s syndrome and abnormalities seen in paraclinical assessments, have been applied extensively for differential diagnosis of superior sulcus tumors. Chest x-ray, computed tomography (CT), whole-body positron emission tomography (PET) and magnetic resonance imaging (MRI) techniques are among the types of imaging examination used for diagnosing and staging Pancoast tumors.^6^ However, there have been almost no reports on the efficiency of whole-body bone scans (WBBS) for detecting the level of abnormality in cases of superior sulcus tumors. Here, we describe a case of Pancoast tumor that showed multiple areas of abnormalities on WBBS, using planar and single-photon emission-computed tomography (SPECT) views.

## CASE REPORT

A 67-year-old male subject with severe hemithorax pain and dyspnea, along with disseminated pain and muscle weakness in the right shoulder and down the arm, was referred to our institution. Pulmonary evaluation revealed coarse as well as dull breathing sounds. Laboratory data showed normocytic normochromic anemia, whereas white blood cells, platelets, liver and renal functions, calcium, phosphorus and alkaline phosphatase (ALP) were all within normal levels and there were no signs of organomegaly or lymphadenopathy. The chest x-ray showed collapse of the right upper lobe. The patient was further evaluated for suspected bronchogenic cancer with brachial plexus involvement. Histopathological analysis showed poorly differentiated squamous cell carcinoma.

**Figure 1.**
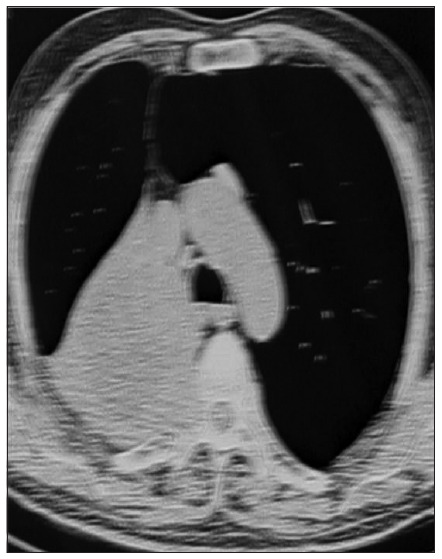
Chest computed tomography with contrast: a mass is seen in the right lung apex with destruction of the adjacent rib vertebra and extension to the spinal canal. Collapse of the right upper lobe is also seen, as a result of bronchial obstruction.

**Figure 2.**
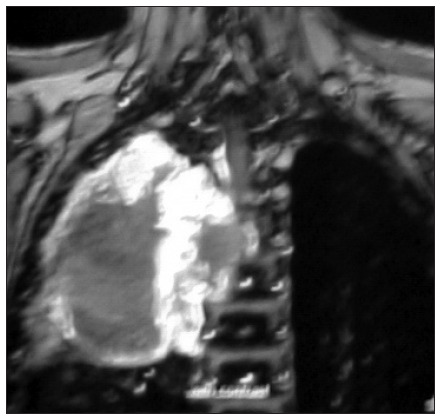
Chest T1-magnetic resonance imaging with gadolinium: a large mass with heterogeneous enhancement of solid components and necrotic non-enhancing component is seen in the right upper lobe, with destruction of vertebrae and invasion into the spinal canal, thereby compressing the spinal cord.

**Figure 3.**
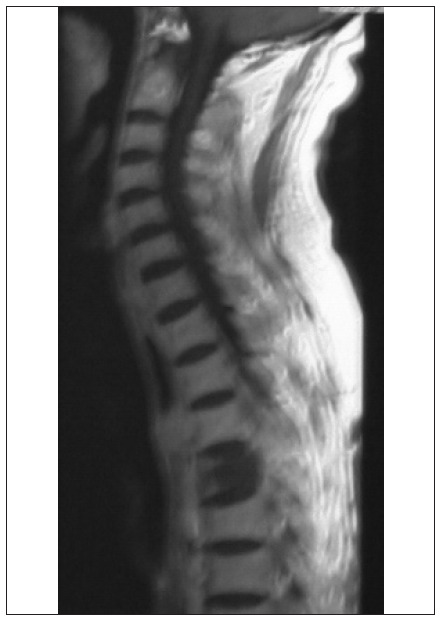
Spine T1-magnetic resonance imaging: a lytic lesion is seen in the T4 vertebral body, suggestive of metastasis from lung carcinoma.

CT of the chest, upper abdomen and brain was performed with contrast and revealed some pulmonary nodules with hilar mass, resorptive atelectasis and obstructive pneumonia, along with destruction of the adjacent rib vertebra and extension to the spinal canal; there were no signs of brain involvement (Figure 1). MRI on the chest also demonstrated destruction of vertebrae and invasion into the spinal canal, thereby compressing the spinal cord (Figure 2). MRI on the spine confirmed the presence of a lytic lesion in the T4 vertebral body (Figure 3).

**Figure 4.**
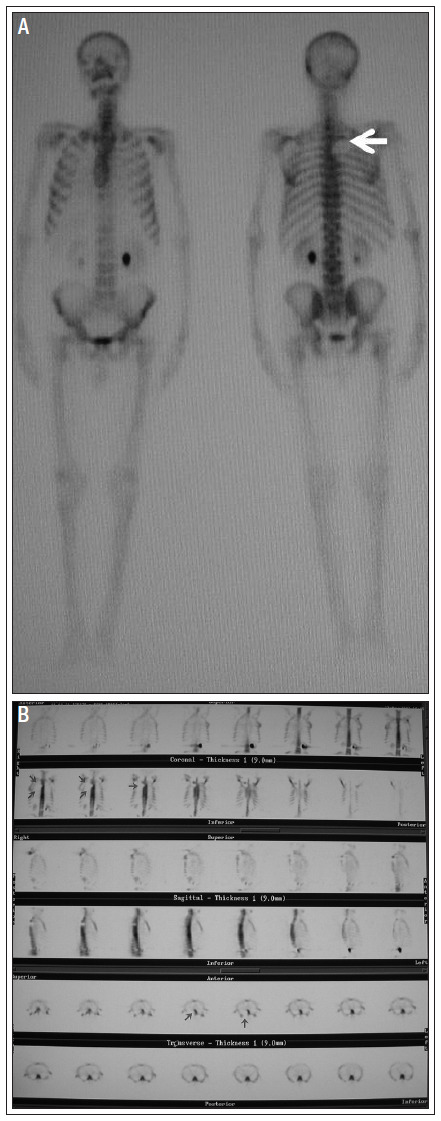
Whole body bone scan (A) in single-photon emission-computed tomography mode (B), revealing areas of decreased radiotracer uptake in the T3-T5 vertebrae and posterior arch of the third to fifth right ribs.

Three hours after administration of 750 MBq (20 mCi) technetium-99m methylene diphosphonate (Tc-99m MDP) by injection, WBBS was performed using a rotating digital gamma camera (ADAC Pegasys) equipped with a low-energy all-purpose parallel whole collimator, with a 20% window centered at 140 keV to provide energy discrimination. SPECT images were obtained in a 128 × 128 matrix, in 64 steps, with 40 s per step. The images were reconstructed and displayed on all three axes: vertical long axis, horizontal long axis and axial short axis. We found an area of diminished radiotracer uptake in the T3-T5 vertebrae and in the posterior arch of the third to fifth right ribs (Figure 4).

**Figure 5.**
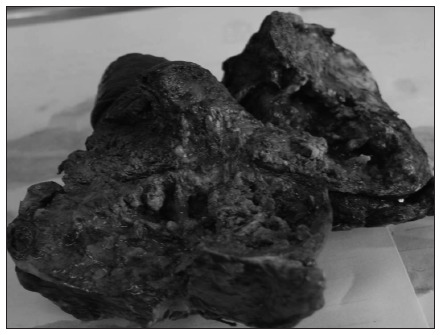
The resected Pancoast tumor.

The patient was further evaluated for induction chemoradiotherapy and was subsequently subjected to a palliative en-bloc surgical operation with a posterior approach (Figure 5).

## DISCUSSION

Assessment of patients with Pancoast tumors is mandatory not only in order to provide a prognosis, but also in order to choose the appropriate treatment approach, so that the morbidity associated with superior sulcus tumors can be minimized.^7^

CT scans or ultrasound-guided percutaneous transthoracic fine-needle biopsies are considered to be the most sensitive technique for identifying Pancoast tumors, whereas CT scans alone provide information about the extent of the disease and recognition of chest-wall involvement, pulmonary nodules and parenchymal diseases.^7,8^ Heelan et al. investigated 32 sets of MRI and CT examinations and concluded that MRI is a procedure with greater accuracy in terms of detection of tumor invasion due to Pancoast tumors.^9^ MRI provides overall better outcomes, not only in determining tumor scope and its extension to the brachial plexus, subclavian vessels, mediastinal lymph node, spinal canal and vertebral bodies, but also in predicting tumor resectability and making radiosensitivity assessments.^1,7,10,11^

Kubota et al. reported that the C-11 methionine uptake pattern in PET showed that large-cell carcinoma with mediastinal invasion decreased in size following radiotherapy, and this was further confirmed by biopsy. On the other hand, CT showed no noticeable change in tumor dimensions.^12^

18F-fluoro-2-deoxy-glucose (FDG)-PET has also proven to be notably more precise than CT and provides lower negative predictive values, not only in discriminating between benign and malignant lesions, but also in lymph node staging.^13,14^ Furthermore, it has been shown that Tc-99m methoxy isobutyl isonitrile (MIBI) SPECT faithfully identifies not only the mediastinal lymph node metastases in patients with NSCLCs,^15^ but also the chemotherapy response in both small-cell lung carcinoma (SCLC)^16^ and NSCLCs.^17^

The functional imaging mode of SPECT has a capability to visualize and measure molecular processes *in vivo*. However, it suffers from low spatial resolution, in comparison with CT or MRI. One way to improve the diagnostic accuracy of this mode, despite its shortcomings, is to combine the images from this mode with images from CT and MRI.^18^

By integrating hybrid systems within one gamma camera, the anatomical inaccuracies due to image fusion would be expected to be greatly reduced, compared with software-based image fusion, although inaccuracies in the thorax due to respiratory movements would still exist.^19^

A dual-headed SPECT camera with a non-diagnostic CT scanner was also recently shown to improve diagnostic specificity with regard to differentiating benign from malignant lesions in bone tissue.^20^

In searching through the main databases (PubMed, Embase, Lilacs and Cochrane Library), we only found five articles relating to bone scanning on Pancoast tumors (Table 1).^21-25^ These studies, which described whole-body bone scans performed on these tumors, were taken into consideration in the present study. However, none of them gave any specific emphasis to SPECT.

**Table 1.** Database search strategies for application of bone scintigraphy in cases of Pancoast tumor

Database	Search strategies	Results		
PubMed	("Pancoast tumor, SPECT"[MeSH]) OR (superior sulcus cancer, bone SPECT) (Pancoast tumor, bone scintigraphy) (Pancoast tumor, bone scan) OR (superior sulcus cancer, bone scintigraphy) OR (superior sulcus cancer, bone scan)	12 articles found	5 relevant articles	3 cross-sectional studies 2 case reports
Embase	same	0	–	–
Lilacs	same	0	–	–
Cochrane Library	same	0	–	–

Embase = Excerpta Medica Database; Lilacs = Literatura Latino-Americana e do Caribe em Ciências da Saúde; MeSH = Medical Subject Headings.

Recently, some studies have shown that SPECT/spiral-CT considerably improves the diagnostic accuracy of radionuclide bone scanning in staging tumor patients.^26,27^

Bone scintigraphy is not routinely necessary in investigating Pancoast tumors. Bone pain is the most common reason for performing WBBS,^28,29^ which may precede the diagnosis of metastasis. WBBS also enables identification of asymptomatic lesions that may produce early local and distant metastatic lesions. As shown in the present case, adding SPECT mode to the planar views in whole-bone scintigraphy can detect local small metastases. We have described this case of Pancoast tumor with emphasis on the clinical and diagnostic points, in the hope of stimulating a higher degree of suspicion and thereby facilitating appropriate diagnosis and treatment.

## CONCLUSIONS

Here, we highlighted WBBS using technetium-99m methylene diphosphonate (Tc-99m MDP) in SPECT mode. We indicated that it was able to detect multiple areas of abnormality in the vertebrae and rib region that were shown using CT scans and MRI. However, additionally, WBBS made it possible to identify an asymptomatic lesion in these tumors, which led to changes to the management of this case.

From our observations, we recommend that a well-designed clinical trial to investigate the efficacy of Tc-99m MDP WBBS in SPECT mode should be conducted in order to investigate its potential implementation for Pancoast tumor evaluation.
